# The Prevalence, Antibiotic Resistance and Biofilm Formation of *Staphylococcus aureus* in Bulk Ready-To-Eat Foods

**DOI:** 10.3390/biom9100524

**Published:** 2019-09-23

**Authors:** Qi Lin, Honghu Sun, Kai Yao, Jiong Cai, Yao Ren, Yuanlong Chi

**Affiliations:** 1 College of Biomass Science and Engineering, and Healthy Food Evaluation Research Center, Sichuan University, Chengdu 610065, China; linqi421@hotmail.com (Q.L.); yaokai555@126.com (K.Y.); reny@scu.edu.cn (Y.R.); 2 Chengdu Institute for Food and Drug Control, Chengdu 610045, China; caijiong147@126.com

**Keywords:** ready-to-eat food, *Staphylococcus aureus*, antibiotic resistance, resistant gene, biofilm formation capacity, food safety

## Abstract

The prevalence of *Staphylococcus aureus* in 2160 bulk ready-to-eat foods from the Sichuan province of China during 2013–2016 was investigated. The antibiotic resistance and the associated genes, as well as biofilm formation capacity of the *S. aureus* isolates were measured. Furthermore, the relationship between the antibiotic resistance and the resistant genes was discussed. It was found that 54 *S. aureus* isolates were recovered, and their prevalence in meat products, dairy, fruit and vegetables, and desserts were 31 (2.6%), six (3.0%), nine (2.2%) and eight (2.3%), respectively. Most strains (52/54) were resistant to at least one of the antibiotics, and 21 isolates were identified as multidrug-resistant (MDR) *S. aureus*. Three isolates were found to be methicillin-resistant *S. aureus*. Penicillin, erythromycin, clindamycin, tetracycline and inducible clindamycin resistance were determined as the predominant antibiotics, and the isolates with the phenotypic resistance on these five antibiotics were all determined positive for the resistant gene associated. In total, 33 of 54 *S. aureus* isolates showed biofilm formation capacity, including two strong biofilm producers, one moderate and 30 weak ones. Two *S. aureus* isolates with strong biofilm formation abilities showed multi-drug resistance, and one moderate biofilm producer was resistant to two categories of antibiotics.

## 1. Introduction

Ready-to-eat food in bulk (RTEIB food) is one of the main categories of food sold in market. It is popular by consumers for its good flavor, nutrition and convenient processing without heat treatment or with low heat treatment. Due to weak sterilization intensity and lack of packing, RTEIB food could be easily contaminated by microorganisms and chemical hazards during transportation, sale and storage. Remarkably, *Staphylococcus aureus* is considered as one of the main food safety hazards [1]. *S. aureus* is one of important foodborne pathogens and can produce Staphylococcal enterotoxins, which can induce severe symptoms; i.e. nausea, violent vomiting, abdominal cramping and diarrhea [2,3]. This pathogen has been detected from some ready-to-eat foods, such as vegetables salads, cooked noodles, cooked meat and desserts [4,5]. Therefore, investigating the prevalence of *S. aureus* could be of great significance for evaluating the food safety risks of RTEIB foods.

The antibiotic resistance of pathogenic bacteria poses great harm to human health and public safety. As is reported, *S. aureus* with antibiotic resistance has caused foodborne outbreaks [6,7,8]. Therefore, understanding the resistance of *S. aureus* on common antibiotics and factors influencing microbial resistance will help with the prevention and elimination of food-borne, resistant *S. aureus*. The antibiotic resistance of *S. aureus* is generally considered to be associated with specific resistance genes. Jarajreh and Ng found that the resistance of *S. aureus* to erythromycin, clindamycin and inducible clindamycin was mainly due to *ermA* or *ermC* genes [9,10]. Ng found the resistance of *S. aureus* to tetracycline was related to *tetM* and *tetK* genes [9]. Moreover, biofilms may also have an impact on the antibiotic resistance of pathogenic bacteria [11]. A biofilm is a mixed extracellular matrix, which is mainly composed of polysaccharides, proteins and RNA or DNA [12]. Kaplan and Wu found that the bacteria in biofilms exhibited 10 to 1500 times more resistance to antibiotics than free cells [13,14]. Biofilms can prevent the access of antibiotics and improve bacterial resistance [12]. To the best of our knowledge, the prevalence of and antibiotic resistance of *S. aureus* in RTEIB foods in the Sichuan province of China are low. In this study, the prevalence of *S. aureus* in 2160 RTEIB food samples collected from Sichuan province, China during 2013–2016, including meat product, dairy, fruit and vegetables, and desserts, was detected. The antibiotic resistance, resistance genes and biofilm forming ability of *S. aureus* isolated from samples were determined. Furthermore, the relationship between the antibiotic resistance and the resistant genes was discussed.

## 2. Materials and Methods

### 2.1. Sample Collection

A total of 2160 RTEIB food samples, including 1209 meat products, 200 dairy products, 401 fruit and vegetables, and 350 desserts, were collected from Sichuan province, China, from 2013 to 2016. Samples were placed in sterile bags and packed in insulated containers with ice for storage. Samples were transported directly to laboratory for testing within 4 h.

### 2.2. Isolation and Identification of Staphylococcus Aureus

Both the isolation and identification of *S. aureus* were performed as previously described by Wang, with minor modifications [15]. Briefly, RTEIB food was ground, and a 25 g minced sample was placed into a sterilized plastic bag, and manually rinsed in 400 mL of buffered peptone water (BPW, Beijing Land Bridge Technology Ltd, China) for 2 min, ensuring that all surfaces were rinsed. The rinsed powder was then incubated at 37 °C for 24 h. A 5 mL aliquot of pre-enrichment product was transferred to 50 mL of trypticase soy broth (TSB, Beijing Land Bridge Technology Ltd.) containing 7.5% NaCl. After incubation at 37 °C for 24 h, a loop of the culture was streaked onto Baird-Parker agar (BPA, Beijing Land Bridge Technology Ltd.) plates with 5% egg yolk and tellurite. Following incubation at 37 °C for 24 h, one or two presumptive coagulase-positive colonies on each sample were selected and transferred into trypticase soy agar (TSA, Beijing Land Bridge Technology Ltd.) plates with 0.6% yeast extract for further purification. Strains isolated were confirmed using VITEK 2 automatic bacteria identification system (BioMerieux, Lyon, France) and 16S rDNA sequencing by Shenggong Biotechnology Co., Ltd., Shanghai, China.

### 2.3. Antimicrobial Susceptibility Testing 

Antibiotic resistance to 17 common drugs covered 13 antimicrobial categories, as shown in Table 1. These categories were applied for the determinations of multidrug-resistant (MDR) and extensively drug-resistant (XDR) *S. aureus*. Multidrug-resistant (MDR) was defined as acquired resistance to at least one agent in three or more antimicrobial categories, and XDR was resistant to at least one agent in all, but susceptible to two or more antimicrobial categories [16]. The minimum inhibitory concentrations (MICs) of 17 drugs were assessed on VITEK 2 system (BioMerieux) using AST-GP67 test card according to the manufacturer’s instructions. Interpretive breakpoints for susceptibility and resistance were consistent with Clinical and Laboratory Standards Institute guidelines (CLSI) in 2018.

### 2.4. The Detection of Antibiotic Resistant Genes 

DNA was extracted using a bacterial genomic DNA extraction kit (TIANGEN, Beijing, China) according to the manufacturer’s instruction. *S. aureus* isolates were tested by polymerase chain reaction for the methicillin resistance gene (*mecA*) to confirm methicillin-resistant *S. aureus* (MRSA). Other antibiotic resistance genes (*blaZ*, *ermC*, *ermA*, *tetK*, *tetM* and *tetL*) were also investigated. All primer sequences are shown in Table 2. The PCR reactions were performed using 96 Well Thermal Cycler PCR (Thermo Flsher Scientific, Shanghai, China). Each PCR reaction contained 1 µL (10 µM) of forward primer, 1 µL (10 µM) of reverse primer, 25 µL Taq HS Perfect Mix (TaKaRa, Beijing, China) and 1 µL of DNA extract. The final volume was 50 µL, made up by adding sterile water. Polymerase chain reactions were performed using an initial denaturation step 94 °C for 5 s, followed by 35 cycles of 94 °C for 5 s, 55 °C for 25 s and 68 °C for 20 s. The PCR products were stained and electrophoresed in 1.5% agarose gel at 120 V for 20 min.

### 2.5. Biofilm Formation Assays

The biofilm formation ability of the strain tested was estimated using the crystal violet staining method described by Jitendra Patel with minor modifications [20]. Overnight cultures of individual *S. aureus* grown in tryptic soy broth (TSB) were adjusted to 0.5 McFarland units with 1/10 TSB. The suspension was then 1:10 diluted with 1/10 TSB, and 200 µL of the diluted suspension was deposited in a sterile 96-well polystyrene microtiter plate. Growth medium devoid of bacterial inoculum served as a negative control. After 48 h of incubation at 28 °C, 200 µL of culture was completely removed by aspiration and the wells were washed five times with sterile distilled water. The plates were air-dried for 20 min, and 200 µL of crystal violet solution (0.41% *w/v* dye, Phygene, Fujian, China) was added and incubated at an ambient temperature for 20 min. After the plates were further washed and air-dried, 200 µL of 95% ethanol was added to dissolve the crystal violet dye. Biofilm formation capacity was characterized by measuring the optical density at 570 nm (OD570) with a microplate spectrophotometer (BioTek Instruments, Winooski, VT, USA). *S. aureus* strain ATCC 6538 was used as a reference strain. All experiments were carried out in triplicate. The cut-off OD (ODc) was defined as three standard deviations above the mean OD of the negative controls. Strains were classified into four categories: not-at-all biofilm producers when OD/ODc ≤ 1, weak biofilm producers when 1 < OD/ODc ≤ 2, moderate biofilm producers when 2 < OD/ODc ≤ 4, or strong biofilm producers when 4 < OD/ODc [21].

### 2.6. Statistical Analyses

Data analyses were performed using statistical product and service solutions (SPSS) for Windows version 22.0 (IBM company, Armonk, NY, USA).

## 3. Result and Discussion

### 3.1. The Prevalence of Staphylococcus Aureus Separated from Ready-to-Eat Food in Bulk in Sichuan Province, China

A total of 2160 RTEIB foods (meat products, *n* = 1209; dairy, *n* = 200; fruit and vegetables, *n* = 401; desserts, *n* = 350) were collected from Sichuan province, China during 2013–2016. It was found that 42 (1.9%) RTEIB samples were detected positive for *S. aureus*; that is, 24 (2.0%) meat products, four (2.0%) dairy, seven (1.8%) fruit and vegetables, and seven (2.0%) desserts, as shown in Table 3. The occurrence of *S. aureus* was almost the same in meat products, dairy and desserts, and was slightly higher compared with the prevalence in fruit and vegetables. Fifty-four *S. aureus* isolates were recovered. The prevalence of *S. aureus* in meat products, dairy, vegetables and fruit, and desserts was 2.6% (31/1209), 3.0% (6/200), 2.2% (9/401) and 2.3% (8/350), respectively. Yang et al. found that 1.1% (39/3417) of bulk ready-to-eat meat products collected from China in 2016 had *S. aureus* at more than 100 CFU/g (colony-forming units/g), and meat with sauce showed the highest microbial contamination rate of 1.6% (30/1909) compared with other four categories of RTEIB meat products [1]. Harada et al. detected 16 (5.7%) *S. aureus* strains from 282 ready-to-eat foods, including six (6.3%) strains from lightly pickled vegetables; seven (8.0%) from a western-style dessert; and three (3.1%) from ready-to-eat fish and seafood products, retailed from Osaka Prefecture, Japan [22]. Kim, Yun and Rhee found that 6.0% (197/3293) of refrigerated ready-to-eat foods (sushi, kimbab and California rolls) were contaminated with *S. aureus* [23]. Those results suggested that the prevalence of *S. aureus* in RTEIB foods from Sichuan province of China was at a relatively low level compared with that from other regions or food types, which might be related with raw material origins, processing technology and analytical methods. However, this study revealed that the *S. aureus* contamination in RTEIB foods from Sichuan province of China should cause more concern.

### 3.2. Phenotypic Resistance and the Associated Genes of Staphylococcus Aureus Isolates

Antimicrobial susceptibility testing showed that all 54 *S. aureus* isolates were susceptible to vancomycin, tigecycline, linezolid and quinupristin/dalfopristin. Antibiotics resistance associated with product types are shown in Table 4. Forty-nine (90.7%), 25 (46.3%) and 22 (40.7%) of the 54 isolates were resistant to penicillin, erythromycin and clindamycin, respectively, followed by 12 (22.2%) isolates that were resistant to tetracycline. 10 (18.5%) had inducible clindamycin resistance. Only several isolates were resistant to ciprofloxacin (13.0%), gentamicin (13.0%), trimethoprim/sulfamethoxazole (11.1%), levofloxacin (7.4%), moxifloxacin (7.4%), cefoxitin (5.6%), oxacillin (5.6%) and rifampin (1.9%). These results could be due to the fact that antibiotics have been used increasingly for treatment of bacterial diseases in humans and animals. Several antibiotics, especially for β-lactams (penicillin), macrolides (erythromycin) and lincosamide (clindamycin), are generally applied in veterinary medicine [24]. The low resistance rate of the isolates to oxacillin may be due to the fact that oxacillin is not commonly used in pesticides, feed and food raw materials. This finding is quite different from the high resistance (70.3%, 52/74) to oxacillin in clinical samples; oxacillin is the drug indicated for the treatment of infections caused by *S. aureus* [25]. Li et al. isolated 104 *S. aureus* strains from 507 raw chicken from retail markets, and 95 (91.3%) isolates showed resistance to penicillin [26]. A total of 128 *S. aureus* isolates were recovered from 87 ready-to-eat foods collected in Bangladesh, and 100 (78.1%) and 52 (40.6%) isolates were resistant to erythromycin and tetracycline, respectively [27]. Zehra et al. detected 89 *S. aureus* strains in 409 retail meats from Punjab, India. Six (6.7%) and six (6.7%) isolates exhibited resistance to clindamycin and inducible clindamycin, respectively [28]. In addition, 21 (38.9%) isolates were identified as MDR (Table 3), and penicillin-erythromycin-clindamycin (P-E-CM) (*n* = 20), penicillin-erythromycin-clindamycin-inducible clindamycin resistance (P-E-CM-ICR) (*n* = 9), penicillin-erythromycin-clindamycin-gentamicin (P-E-CM-GM) (*n* = 7) and penicillin-erythromycin-clindamycin-trimethoprim/sulfamethoxazole (P-E-CM-SXT) (*n* = 6) were the main MDR profiles in the isolates. MDR isolates were distributed in four types of RTEIB foods, and it was noted that all the six isolates from dairy were determined to be MDR. Three (5.6%) isolates were identified as MRSA, one of which was isolated from a meat product and the other two were from desserts. None that were XDR were detected.

A combined comparison was carried out in four products categories. The *S. aureus* isolates from meat products showed resistance to 10 categories (13 kinds) of antibiotics, and the isolates from dairy, vegetables and fruit, and desserts, were resistant to seven categories (seven kinds), seven categories (nine kinds) and six categories (seven kinds), respectively. This founding could be correlated with the frequent administration of macrolide-class tylosin to animals, resulting in the development of cross-resistance to the Macrolides, Lincosamides and Streptogramins [29], but more meat product samples were tested than the other three foods in this study. It was found that penicillin resistance exhibited high rates in all four products. Erythromycin and clindamycin resistance rates are significantly higher in meat products and dairy compared to fruit, vegetables and desserts. The existence of inducible clindamycin resistance was only found in meat products and dairy. In addition, other antibiotic resistances were randomly distributed because of low performance of resistance.

Penicillin, erythromycin, clindamycin, tetracycline and inducible clindamycin resistance were determined as the predominant antibiotics resisted by the 54 *S. aureus* isolates (as shown in Table 4), and so their resistant genes, i.e. *blaz*, *erm* (*ermA* and/or *ermC*) and *tet* (*tetL*, *tetM* and/or *tetK*), were also measured. In total, 54 (100%), 47 (87%) and 51 (94.4%) of 54 isolates were positive for genes *blaz*, *erm* and *tet*, respectively. It is worth noting that the isolates with the phenotypic resistance od these five antibiotics, were all determined positive for the resistant gene associated, as shown in Table 5. Zelazny reported that *ermA* and *ermC* genes could encode methylase, which was involved in the modulation of resistance against clindamycin and erythromycin [30]. Therefore, a resistance gene can be used as an important reference for the evaluation of microbial resistance. However, the isolates with resistance genes do not always exhibit phenotypic resistance.

### 3.3. The Biofilm Formation Abilities of Staphylococcus aureus Isolates

**Figure 1:**
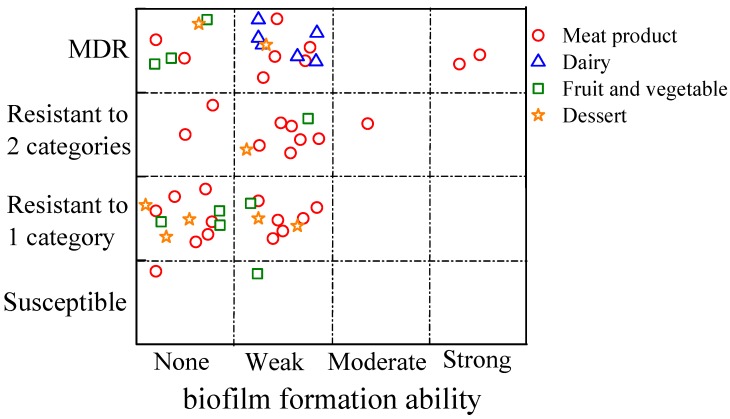
Antibiotic resistance categories and the biofilm formation abilities of the 54 *S. aureus* isolates from RTEIB foods, including meat products, dairy, fruit and vegetables, and desserts. No biofilm formation ability when OD/cut-off OD (ODc) ≤ 1, weak when 1 < OD/ODc ≤ 2, moderate when 2 < OD/ODc ≤ 4, strong when 4 < OD/ODc.

As shown in Table 5, 33 (61.1%) of 54 *S. aureus* isolates showed biofilm formation capacity, including two (3.7%) strong biofilm producers, one (1.9%) moderate and 30 (55.6%) weak. It revealed that most (51/54) of the isolates in this study possessed no or weak biofilm formation ability, which is consistent with the *S. aureus* isolated from foods and is greatly different to that collected from clinical samples. Kroning et al. reported that the *S. aureus* isolates from handmade sweets were all characterized as not-at-all or weak biofilm producers [31]. Bimanand et al. found that 92 (95.8%) of the *S. aureus* isolates from clinical samples were biofilm producers, and the distributions of biofilm formation between isolates were four (4.2%), 52 (54.2%) and 44 (35.4%) as strong, moderate and weak, respectively [32]. Comparison from four products categories: 20 (64.5%) of 31 isolates from meat products had biofilm formation ability, and six (100%), three (33.3%) and four (50%) of the isolates were characterized as biofilm producers from dairy, vegetables and fruit, and desserts, respectively. The isolates from meat products showed varied biofilm formation ability, including two strong biofilm producers, one moderate, 17 weak and 11 not-at-all ones. The six isolates from dairy all had weak biofilm formation capacity, while the strains from fruit and vegetables, and desserts, exhibited weak or no ability. Aslantas and Demir found that the *S. aureus* with biofilm formation ability could be detected in cows, especially in bovine mastitis cases [33], which might be the reason for the high rate of biofilm producers detected from dairy in this study. Antibiotic resistant categories and the biofilm formation ability of the 54 *S. aureus* isolated from RTEIB foods are shown in Figure 1. Two *S. aureus* isolates with strong biofilm formation ability showed multidrug-resistance, and one moderate biofilm producer was resistant to two categories of antibiotics. The weak or not-at-at-all biofilm producers showed different antibiotic resistance ranges, from one to three or more categories, except two isolates.

## 4. Conclusions

Fifty-four *S. aureus* isolates were recovered from 2160 samples, and their presence in meat products, dairy, fruits and vegetables and desserts were 31/1209 (2.6%), 6/200 (3.0%), 9/401 (2.2%) and 8/350 (2.3%), respectively. Most strains (52/54) were resistant to at least one of the antibiotics, and 21 (38.9%) and three (5.6%) isolates were respectively identified as MDR and MRSA. These results suggested that the contamination and antibiotic resistance of *S. aureus* in RTEIB foods from the Sichuan province of China should cause more concern. The *S. aureus* isolates with the phenotypic resistance on the five predominant antibiotics; i.e., penicillin, erythromycin, clindamycin, tetracycline and inducible clindamycin resistance, were all determined positive for the resistance gene associated, and targeting the resistance gene can thus be used as an important reference for the evaluation of microbial resistance. Thirty-three (61.1%) *S. aureus* isolates were characterized as biofilm producers. 

## Figures and Tables

**Table 1:** Antimicrobial categories and agents used in this study.

Antimicrobial Category	Antimicrobial Agent
Penicillins	Penicillin
Aminoglycosides	Gentamicin
Ansamycins	Rifampin
Anti-staphylococcal β-lactams	Oxacillin
	Cefoxitin
Fluoroquinolones	Ciprofloxacin
	Levofloxacin
	Moxifloxacin
Folate pathway inhibitors	Trimethoprim/sulphamethoxazole
Glycopeptides	Vancomycin
	Tigecycline
Macrolide	Erythromycin
Lincosamide	Clindamycin
Oxazolidinones	Linezolid
Streptogramins B	Quinupristin/dalfopristin
Tetracyclines	Tetracycline
Macrolide-Lincosamide-Streptogramin B	Inducible Clindamycin Resistance

**Table 2:** Antibiotic resistant genes and primers used in this study.

Antibiotics	Target Gene	Primer Sequence (5’-3’)	Size (bp)	Reference
Penicillin	*blaZ*	F: CAAAGATGATATAGTTGCTTATTC	355	[17]
		R: CATATGTTATTGCTTGCACCAC		
Cefoxitin Oxacillin	*mecA*	F: AACAGGTGAATTATTAGCACTTGTAAG	173	[18]
		R: ATTGCTGTTAATATTTTTTGAGTTGAA		
Inducible clindamycin resistance Erythromycin Clindamycin	*ermA*	F: GTTCAAGAACAATCAATACAGAG	421	[19]
		R: GGATCAGGAAAAGGACATTTTAC		
	*ermC*	F: GCTAATATTG TTTAAATCGT CAATTCC	572	
		R: GGATCAGGAAAAGGACATTTTAC		
Tetracycline	*tetL*	F: TCGTTAGCGTGCTGTCATTC	267	[9]
		R: GTATCCCACCAATGTAGCCG		
	*tetM*	F: GTGGACAAAGGTACAACGAG	406	
		R: CGGTAAAGT TCG TCACACAC		
	*tetK*	F: TCGATAGGAACAGCAGTA	169	
		R: CAGCAGATCCTACTCCTT		

**Table 3:** The prevalence of *Staphylococcus aureus*—multidrug-resistant (MDR), extensively drug-resistant (XDR) and methicillin-resistant *S. aureus* (MRSA) types in 2160 RTEIB foods, including meat, dairy, fruit and vegetables, and desserts.

Types of RTEIB	Total No. of Samples		Detection of *S. aureus*			
		No. (%) of Samples	No. *S. aureus*	No. MDR ^a^	No. XDR ^b^	No. MRSA
Meat product	1209	24 (2.0)	31	10	0	1
Dairy	200	4 (2.0)	6	6	0	0
Fruit and vegetable	401	7 (1.8)	9	3	0	0
Dessert	350	7 (2.0)	8	2	0	2
Total	2160	42 (1.9)	54	21	0	3

^a^ MDR is defined as acquired resistance to at least one agent in three or more antimicrobial categories. ^b^ XDR is defined as resistance to at least one agent in all but just susceptibility to two or fewer antimicrobial categories.

**Table 4:** Antimicrobial resistance profiles of *S. aureus* isolates from Ready-to-eat food in bulk (RTEIB foods), including meat, dairy, fruits and vegetables, and desserts.

Source *	Antimicrobial Resistance Profiles **												
	P	E	CM	TE	ICR	CIP	GM	SXT	LEV	MXF	FOX	OX	RD
Meat product	27	14	11	8	7	4	2	2	3	3	1	1	1
Dairy	6	6	6		3	2	3	2					
Fruit and vegetable	8	3	3	1		1	1	2	1	1			
Dessert	8	2	2	3			1				2	2	
Total (54 isolates)	49	25	22	12	10	7	7	6	4	4	3	3	1

* All 54 isolates were susceptible to vancomycin, tigecycline, linezolid and quinupristin/dalfopristin, and the data is not shown. ** P, penicillin; E, erythromycin; CM, clindamycin; TE, tetracycline; ICR, inducible clindamycin resistance; CIP, ciprofloxacin; GM, gentamicin; SXT, trimethoprim/sulfamethoxazole; LEV, levofloxacin; MXF, moxifloxacin; FOX, cefoxitin; OX, oxacillin; RD, rifampin.

**Table 5:** Phenotypic resistance (P) and the associated genes (G), as well as the biofilm formation abilities of 54 *S. aureus* isolates from RTEIB foods, including meat, dairy, fruit and vegetables, and desserts.

	Penicillin		Erythromycin		Clindamycin		Tetracycline		Inducible Clindamycin Resistance		Biofilm Formation Ability **
	P	G (*blaZ*)	P	G (*erm*) *	P	G (*erm*) *	P	G (*tet*) *	P	G (*erm*) *	
**Meat product**											
MT01	R ***	+	R	+	R	+	R	+		+	None
MT02	R	+	R	+	R	+		+	R	+	Strong
MT03	R	+	R	+	R	+		+	R	+	Strong
MT04	R	+	R	+	R	+		+		+	Weak
MT05	R	+	R	+	R	+	R	+	R	+	Weak
MT06	R	+	R	+	R	+		+	R	+	None
MT07	R	+	R	+	R	+		+	R	+	Weak
MT08	R	+	R	+	R	+		+	R	+	Weak
MT09	R	+	R	+	R	+		+		+	None
MT10		+	R	+	R	+		+	R	+	Weak
MT11	R	+	R	+		+	R	+		+	Weak
MT12	R	+		+		+				+	None
MT13		+	R	+	R	+		+		+	None
MT14	R	+		+		+	R	+		+	Moderate
MT15	R	+		+		+	R	+		+	Weak
MT16	R	+		+		+	R	+		+	Weak
MT17	R	+					R	+			Weak
MT18	R	+	R	+		+	R	+		+	Weak
MT19	R	+	R	+		+		+		+	Weak
MT20		+		+		+	R	+		+	Weak
MT21	R	+		+		+		+		+	Weak
MT22	R	+		+		+		+		+	None
MT23	R	+		+		+		+		+	Weak
MT24	R	+		+		+		+		+	None
MT25	R	+		+		+		+		+	Weak
MT26	R	+		+		+		+		+	None
MT27	R	+						+			None
MT28	R	+						+			Weak
MT29	R	+						+			Weak
MT30	R	+									None
MT31	R	+		+		+		+		+	None
**Dairy**											
DY01	R	+	R	+	R	+		+		+	Weak
DY02	R	+	R	+	R	+		+		+	Weak
DY03	R	+	R	+	R	+		+		+	Weak
DY04	R	+	R	+	R	+		+	R	+	Weak
DY05	R	+	R	+	R	+		+	R	+	Weak
DY06	R	+	R	+	R	+		+	R	+	Weak
**Fruit and vegetable**											
FV01	R	+	R	+	R	+		+		+	None
FV02	R	+	R	+	R	+		+		+	None
FV03	R	+	R	+	R	+		+		+	None
FV04	R	+		+		+		+		+	None
FV05	R	+		+		+	R	+		+	Weak
FV06	R	+		+		+		+		+	Weak
FV07	R	+		+		+		+		+	None
FV08	R	+									None
FV09		+		+		+		+		+	Weak
**Dessert**											
CY01	R	+	R	+	R	+	R	+		+	Weak
CY02	R	+	R	+	R	+	R	+		+	None
CY03	R	+		+		+	R	+		+	None
CY04	R	+		+		+		+		+	Weak
CY05	R	+						+			Weak
CY06	R	+		+		+		+		+	Weak
CY07	R	+		+		+		+		+	None
CY08	R	+		+		+		+		+	None

* Genes *erm* include *ermA* and/or *ermC*; genes *tet* include *tetL*, *tetM* and/or *tetK*. ** No biofilm formation ability means OD/ODc ≤ 1, weak—1 < OD/ODc ≤ 2, moderate 2 < OD/ODc ≤ 4, strong (4 < OD/ODc). *** R represents resistance, and + represents detected.
